# Bird Diversity and Distribution in relation to Urban Landscape Types in Northern Rwanda

**DOI:** 10.1155/2014/157824

**Published:** 2014-07-15

**Authors:** T. Gatesire, D. Nsabimana, A. Nyiramana, J. L. Seburanga, M. O. Mirville

**Affiliations:** ^1^University of Rwanda, P.O. Box 117, Butare, Rwanda; ^2^Karisoke Research Center, Dian Fossey Gorilla Fund International, 800 Cherokee Avenue Southeast, Atlanta, GA 30315-1440, USA

## Abstract

Using the point count method, linear mixed models, Shannon's diversity index, and Bray-Curtis cluster analysis, we conducted a study of the effect of urban fabric layout on bird diversity and distribution in northern Rwanda. The results showed a significant effect of city landscapes on bird richness and relative abundance; residential neighborhoods, institutional grounds, and informal settlements had the highest species diversity in comparison to other microlandscape types. Riversides were characterized by specialized bird species, commonly known to be restricted to wetland environments. Built-up areas and open field landscapes had comparable results. One Albertine Rift endemic bird species, the Ruwenzori Double-collared Sunbird (*Cinnyris stuhlmanni*), was recorded. Three migratory birds were found in Musanze city for the first time: the Common Sandpiper (*Actitis hypoleucos*), the Spotted Flycatcher (*Muscicapa striata*), and the Willow Warbler (*Phylloscopus trochilus*). Two bird species have not been previously reported in Rwanda: the Garden Warbler (*Sylvia borin*) and the Lesser Spotted Eagle (*Aquila pomarina*). The implications of this study are particularly relevant to urban decision makers who should consider the existence of a great diversity of avian fauna when developing and implementing master plans, especially when villages and cities are in proximity of protected areas or natural reserves.

## 1. Introduction

By the year 2050, it is estimated that the majority of the global population will live in urban areas [1]. Threats to biodiversity are particularly inherent to such rapid urbanization, which raises concern over the future of the already reduced diversity in settings surrounding urban neighborhoods [2]. In many developing countries, a large number of wildlife survive outside protected areas on farmlands, pasturelands, and in urban areas [3, 4]. Among all wildlife, birds are one of the most common wildlife in urban areas such as neighborhoods and cities, and many bird populations have been declining as a result of landscape changes due to urban expansion [5–8]. At the local level, these major changes include high rates of land conversion into urban uses and increasing human pressure on biodiversity due to rapid population growth.

Due to the important role that birds play in maintaining ecosystems and supporting biodiversity, many seek their protection to manage biological threats and efficiently protect the environment [9].

Birds fulfill many ecological functions in their habitats. For instance, they are bioindicators of healthy ecosystems [10, 11]. In addition, insectivorous species and raptors regulate disease vectors, including mosquitoes and rodents. Scavenger birds, such as the Pied Crow (*Corvus albus*), contribute to biomass recycling and to some degree reduce levels of disposable wastes. Frugivorous birds play an important role in seed dispersal of fleshy fruit-producing plants [9]. Birds are also important in plant pollination as demonstrated by sunbirds, which participate in crossbreeding of flowering plants, especially those with bird-pollination syndrome [12]. These ecosystem services are important for many communities, and to ensure that birds can fulfill these biological roles at an appropriate level for current and future generations, there is a pressing need to study the dynamics and socioeconomics of bird diversity outside protected areas, especially in urban areas.

The landscape of the Musanze District in the Northern Province of Rwanda has been undergoing major changes due to rapid urbanization driven by a fast growing human population [13]. Human density in this region can reach up to 1,000 people per km^2^ [14] and it is estimated that more than half of the total Rwandan population is expected to live in urban areas in less than 40 years [15]. High rates of land conversion into urban use in Musanze is threatening the wild avian diversity, as it is the case for other fast growing cities in the world [16]. More than half of the total Rwandan population is expected to live in urban areas in less than 40 years [15].

While wild avian diversity has been a subject of research in natural habitats of the Northern Province, especially in the Parc National des Volcans (PNV) and the Buhanga eco-park, the biodiversity of the neighboring Musanze city, like many other anthropogenic landscapes, remains understudied. Thus, this study aimed to address the issue of ecological bias in bird diversity and distribution in fast growing cities and proposes strategies for effective conservation of birds in urban areas of Rwanda and of Musanze city, in particular. The main objective of this study was to assess the diversity and distribution of birds in urban landscape types of the Musanze city. Specifically, the study aimed (1) to identify bird species that live in or visit Musanze city, (2) to identify bird feeding guilds, bird abundance, and bird diversity in different landscapes in Musanze, (3) to locate areas of the city that show high bird diversity, hereafter referred to as “urban bird hotspots,” and (4) to compare the bird diversity in Musanze city with the bird diversity found in the PNV and Buhanga eco-park, which are two important ecological zones in the administrative district where Musanze city is located. Our study therefore provides policy makers and conservationists with scientific information about the ecological status of birds in Musanze city and how species are distributed in the city in relation to the urban fabric layout.

## 2. Materials and Methods

### 2.1. Study Area

Musanze District is situated in the northwestern highlands of Rwanda and is one of the five administrative districts within the Northern Province. The study was carried out within Musanze city, which covers a total area of 61.97 km² and is comprised of four sectors: Muhoza, Cyuve, Musanze, and Kimonyi. Musanze is one of the largest and fastest growing urban centers in Rwanda and is a central hub for businesses, trade, and tourism. The PNV with its famous mountain gorillas, the Buhanga eco-park, and the Ruhondo Lake are found in Musanze and make the city a popular destination for national and international tourists (http://www.musanze.gov.rw, 30.12.2012).

### 2.2. Landscape Stratification

Landscapes of Musanze city can be classified into two major categories: open fields and built-up areas. Open fields can include agricultural land, cemeteries, wastelands, stream banks, forests, and the aerodrome, which is a nonpaved strip covered by a regularly mown lawn of about 1.5 km length. Built-up areas include business centers, institutional grounds, settlements, and residential neighborhoods.

In this study, agricultural land referred to cultivated lands located within Musanze surrounding urban areas, where maize and beans are the most common crops. We included two cemeteries in our sampling sites, which were located in the Muhoza and Cyuve sectors. Wastelands were defined as areas dedicated to household solid waste disposal. Two wastelands were considered for the study, one close to Musanze business center and another in Cyuve. We considered stream banks as land that encompassed habitats on both sides of permanent or seasonal river flows. Forests included areas covered by tree plantations, mainly Eucalypts. Natural forests were not found within the city boundaries. The Musanze aerodrome is a nonpaved strip covered by a regularly mown lawn of about 1.5 km length.

Business centers included market places and areas with a high concentration of shops. Institutional grounds were comprised of large institution gardens, including the district office, Musanze hospital, one university, one high school, and the city abattoir. Informal and nonformal settlements included organized village settlements and rural type scattered settlements, where banana plantations were the most common crop in the home gardens. Residential neighborhoods were high standard settlement areas of the city.

### 2.3. Data Collection

During March 2012, data were collected within plots using the point count method [17]. The point count sampling design consisted of a series of points at which birds were counted within a defined radius [18]. Forty 50 m radius plots were established using a global positioning system (GPS) unit; twenty in open fields and other twenty in built-up areas (Table 1). Between two and five plots were randomly selected for each microlandscape type (Table 1) within a 0.4 × 0.4 km grid at a rate of one plot per grid cell. Each plot was visited four times for a ten-minute observation period [19], such that this study included 160 plot visits in total. Visits to each plot were equally distributed across four periods during the day: early morning (6:30–8:30), late morning (9:00–11:00), early evening (15:00–17:00), and late evening (17:30–18:30). As the weather can influence the occurrence of some bird species [20], working during rain or strong wind was avoided.

In addition to a presurvey training of data collectors on bird identification, a bird expert was present in 96% of our field visits. A pair of 8 × 42 resolution binoculars (Olympus mark) and a field guide book were also used to identify the species of birds observed [9]. Five minutes after the plot was located, the center was chosen whenever possible to observe any bird movement. This time delay was in place to allow birds to return to their natural behavior in order to minimize potential impacts of human presence on the survey. The following variables were recorded during each 10 min observation period: number of plot, type of microlandscape, arrival time, departure time, species names of birds observed, number of individuals from each observed bird species, weather conditions, vegetation cover, and any kind of disturbance, such as extra human presence or noise from nearby vehicles.

Birds encountered outside of our study plots were recorded only when it was a locally novel species, such that it had never been observed in the area before. These records were only considered for compiling a bird checklist of Musanze city and were therefore excluded from statistical analysis. When a bird could not be identified in the field, photos from a high-resolution camera and descriptive notes were taken for later identification by a bird expert.

### 2.4. Data Analysis

The linear mixed models (LMM) method was used to analyze the relationships between landscape, bird abundance, and species richness. LMM recently became a useful tool used to analyze continuous repeated measures data from a sample of individuals in different areas [21]. LMM is a statistical model that consists of both fixed effects and random-effects terms and was therefore the most appropriate choice for our study [22]. In this study, LMM was used to assess whether urban landscape types have an effect on bird abundance and species richness. Landscape types (macro- and microlandscapes) were fixed effects (covariates), while sampling plots were treated as a random effect to control for repeated measures in plots. Bird species richness and relative abundance were dependent variables assessed in different LMMs.

The Shannon's diversity index (*H*′ = −∑*p*_*i*_log⁡*p*_*i*_, where *p*_*i*_ is the proportion of individuals belonging to the *i*th species) was used to identify the *α*-diversity of bird species both at city and landscape type levels in order to identify the local average species diversity of each particular habitat. The Bray-Curtis cluster analysis (single link) method was used to assess the level of similarity in bird composition between landscape types [23].

Referring to Tuyisingize and Fawcett [24], from which we retrieved the checklist of birds of the PNV and Buhanga eco-park, the bird diversity and abundance of Musanze city were compared with the two natural ecosystems of these parks in terms of bird species richness and composition using the hierarchical clustering method.

**Figure 1:**
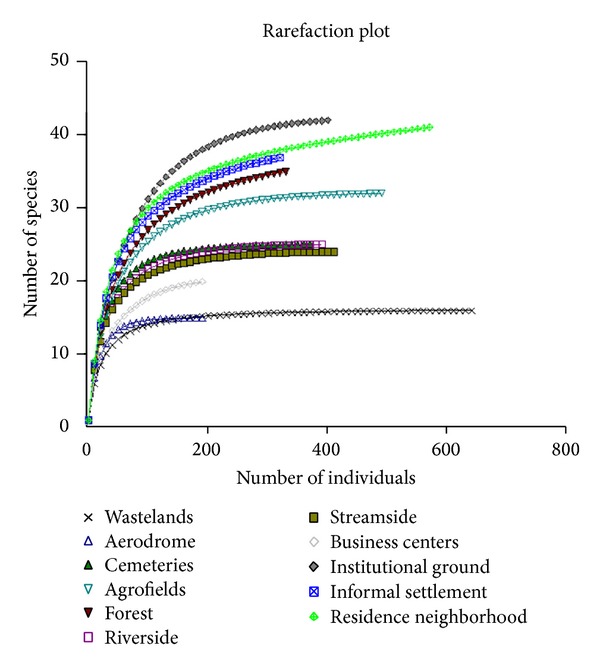
Bird species rarefaction curves at the site considered in this study.

To examine the relationship between bird species and food availability in the city, bird species were categorized according to their feeding guilds. The completeness of the survey was assessed by analyzing patterns of the species accumulation curves [25]. The accumulation curves of species richness in different microlandscape types globally reached their plateau (Figure 1), giving us confidence to conclude that our sampling efforts had covered the majority of species in Musanze.

To determine the level of significance of the obtained results, hypothesis testing tools were used, including the test of Student's *t*-test. Bioprofessional, MVSP, Origin and R software packages were used for these analyses.

## 3. Results

### 3.1. Species Richness

A total of 94 bird species were observed in Musanze city, 15 of which were found opportunistically outside plots. Only one Albertine Rift endemic species (Ruwenzori Double-collared Sunbird,* Cinnyris stuhlmanni*) was observed, and seven migrant bird species were recorded.

#### 3.1.1. Number of Bird Species by Macrolandscape Types

**Figure 2:**
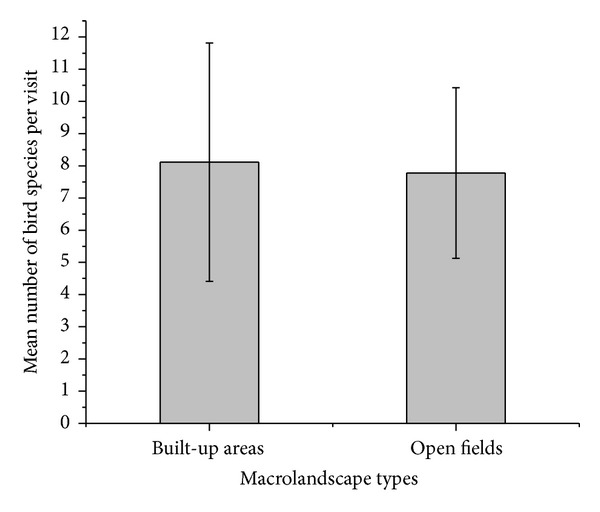
Mean number of species observed during plot visits in the two macrolandscape types.

There was no significant difference in the number of bird species in open fields and built-up areas (*t* = −0.42; *P* = 0.67; Figure 2). Similarly, the total number of bird species observed in both macrolandscapes was not notably different (*N* built-up = 63, *N* open fields = 61).

#### 3.1.2. Number of Species by Microlandscape Types

**Figure 3:**
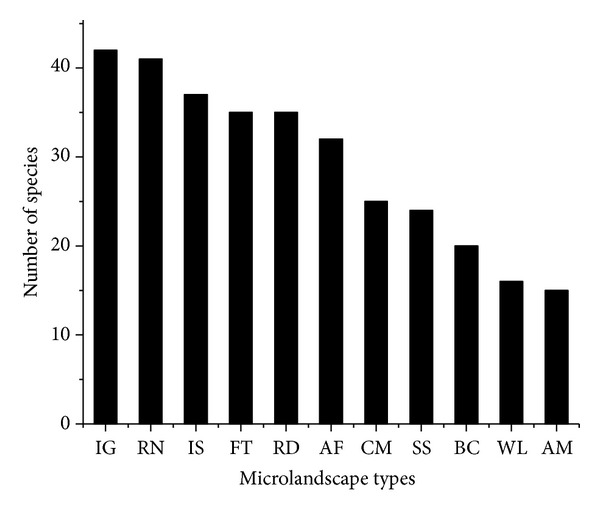
Total bird species in microlandscape types (AM: aerodrome; AG: agrofields; BC: business centers; CM: cemeteries; FT: forests; IS: informal settlements; IG: institutional grounds; RN: residential neighborhoods; RS: riversides; SS: streamside; WL: wasteland).

Among the different microlandscapes, institutional grounds had the highest number of species (*N* = 42), followed by residential neighborhoods (*N* = 41) and informal settlements (*N* = 37). Furthermore, forest and riverside landscapes had similar number in terms of total number of species (*N* = 35 for both). Finally, wastelands and the aerodrome demonstrated the smallest number of bird species (*N* = 16 and *N* = 15, resp.) (Figure 3).

**Figure 4:**
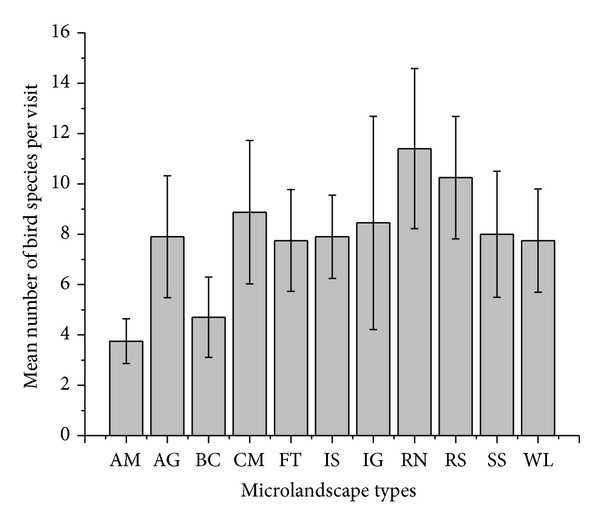
Mean number of bird species observed during plot visits in each microlandscape type (AM: aerodrome; AG: agrofields; BC: business centers; CM: cemeteries; FT: forests; IS: informal settlements; IG: institutional grounds; RN: residential neighborhoods; RS: riversides; SS: streamside; WL: wasteland).

For the LMM that examined the impact of different microlandscapes on the number of bird species, residential neighborhoods were used as the reference microlandscape type as it showed the highest average number of bird species per plot visit (Figure 4). Thus, the average number of bird species found in any other microlandscape was compared to the number of bird species found in residential neighborhoods. The LMM analysis revealed that the average number of bird species found in all microlandscapes, excluding cemeteries and riversides, was significantly lower than the number of bird species in residential Musanze's neighborhood areas (Table 2). The average number of bird species observed in plots located in cemeteries tended to be less than the average number found in residential neighborhoods.

### 3.2. Species Relative Abundance

#### 3.2.1. Number of Individuals by Macrolandscape Types

**Figure 5:**
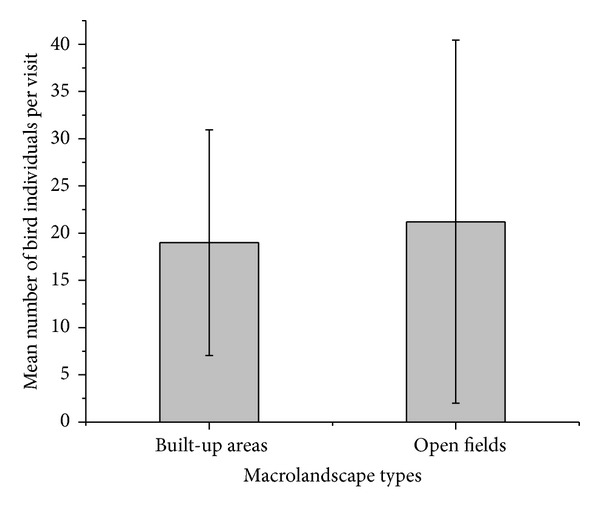
Mean number of bird individuals encountered per visit by macrolandscape type.

There was no significant difference between open fields and built-up areas in terms of number of bird individuals observed per visit (*t* = 0.35; *P* = 0.73; Figure 5).

#### 3.2.2. Number of Individuals by Microlandscape Types

**Figure 6:**
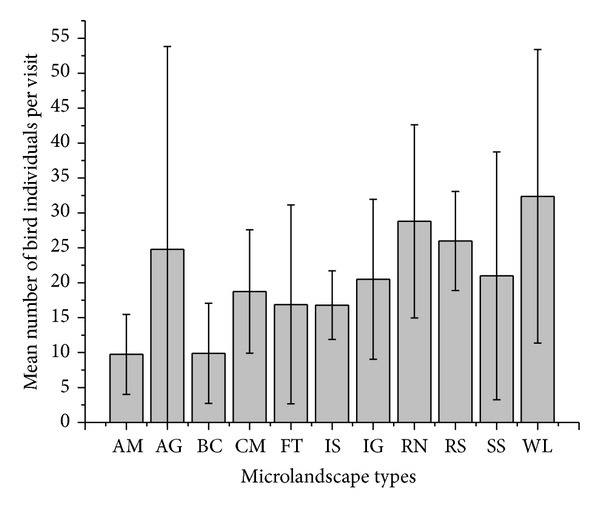
Mean number of bird individuals observed per plot visit by microlandscape type (AM: aerodrome; AG: agrofields; BC; business centers; CM; cemeteries; FT: forests; IS: informal settlements; IG: institutional grounds; RN: residential neighborhoods; RS: riversides; SS: streamside; WL: wasteland).

There were significantly less birds in all microlandscape types compared to the number of birds encountered in plots located in residential neighborhoods, except for cemeteries (where fewer birds were observed), riversides, and wastelands (Figure 6 and Table 3).

#### 3.2.3. Most Represented Species

**Figure 7:**
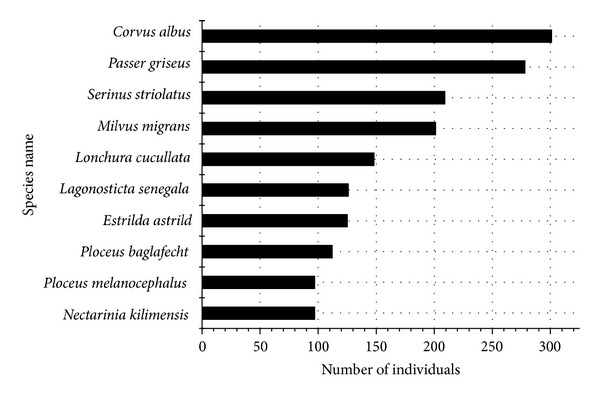
Top ten most represented bird species in all sites of Musanze city.

The Pied Crow (*Corvus albus*), the Grey-headed Sparrow (*Passer griseus*), Streaky Seedeater (*Serinus striolatus*), and the Black Kite (*Milvus migrans*) were found to be the most abundant species in this study (Figure 7).

### 3.3. Feeding Guild Categories

#### 3.3.1. Feeding Guild Categories by Macrolandscape Types

**Figure 8:**
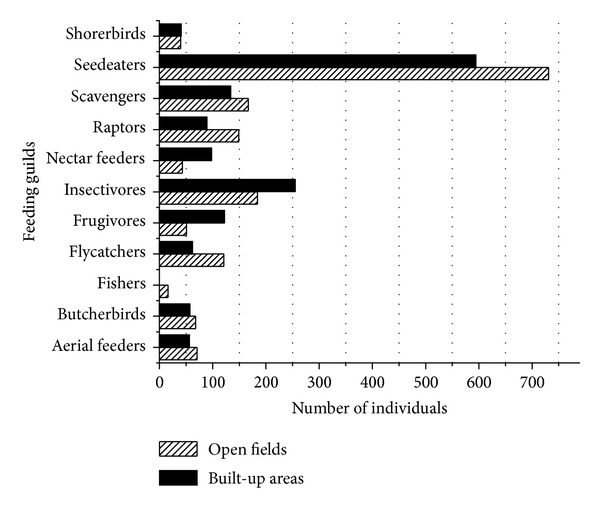
Feeding guild categories of bird species as classified in microlandscapes types.

Among the eleven feeding guilds represented by bird species in Musanze city, seedeaters were the most common in both built-up areas and open fields, followed by insectivorous species and scavengers (Figure 8).

#### 3.3.2. Feeding Guild Categories by Microlandscapes

Seed-eating bird species were the most common across all microlandscapes except for wastelands, which were dominated by scavengers, a feeding type that was also recorded in every landscape. Insectivorous species were also present in all microlandscape types, unlike fishers that were only recorded in riverside landscapes. Riverside landscapes were also where flycatchers, a feeding type that was present in all landscape types, were most abundant.

### 3.4. Species Diversity

#### 3.4.1. Alpha Diversity in Macro- and Microlandscape Types

**Figure 9:**
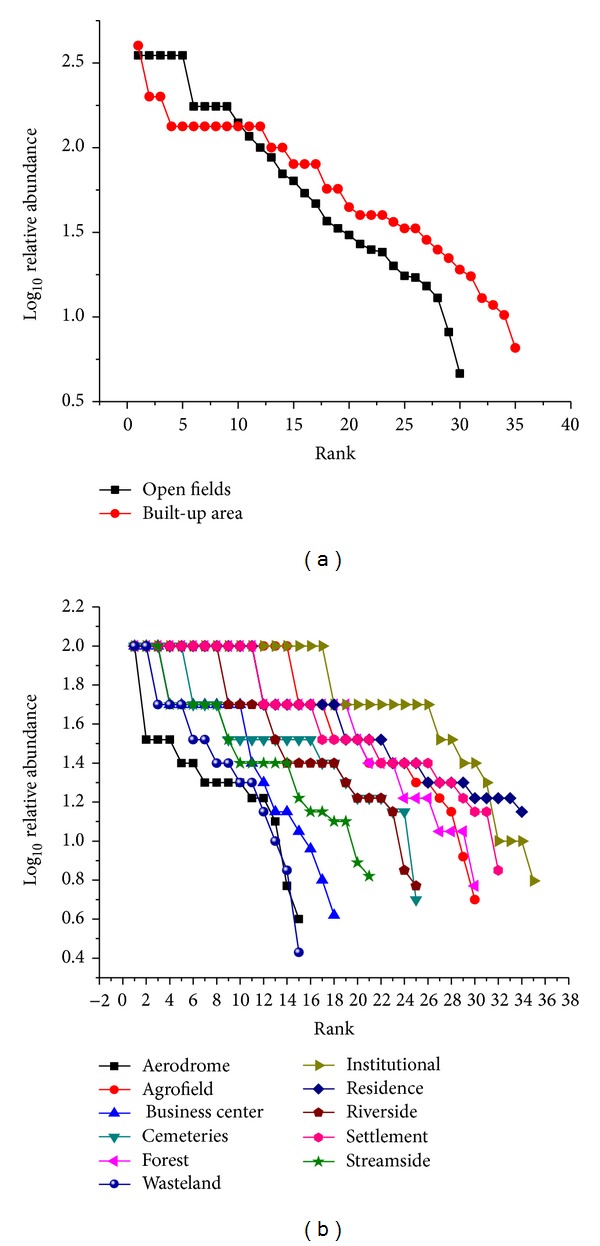
Species rank/abundance: (a) in macrolandscape types and (b) in microlandscape types.

The bird species diversity in both macro- and microlandscapes of Musanze are described using Whittaker curves (Figure 9). Using these curves, built-up areas were slightly higher ranked in terms of bird diversity than open fields (Figure 9(a)). Among microlandscapes, institutional grounds had the highest diversity rank, followed by residential neighborhoods and informal settlements. The aerodrome and Westland were ranked with the lowest bird diversity (Figure 9(b)).

**Figure 10:**
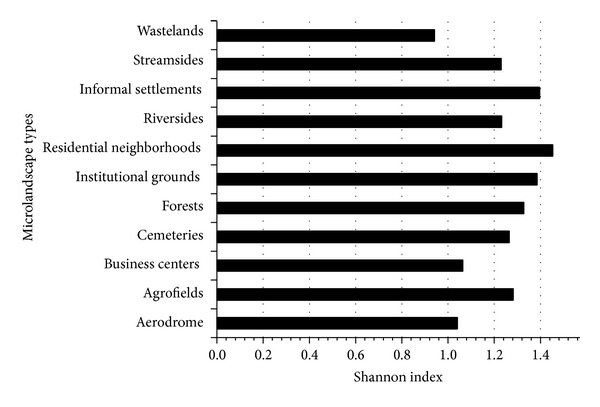
Shannon index for microlandscape types of Musanze city.

The Shannon diversity index was similar for both macrolandscape types, built-up areas (*H*′ = 1.456) and its counterpart, open fields (*H*′ = 1.518). At the microlandscape level, residential neighborhoods were the most diverse, followed by informal settlements and institutional grounds. Wastelands displayed the lowest bird diversity (Figure 10).

#### 3.4.2. Species Similarity in Macro- and Microlandscape Types

**Figure 11:**
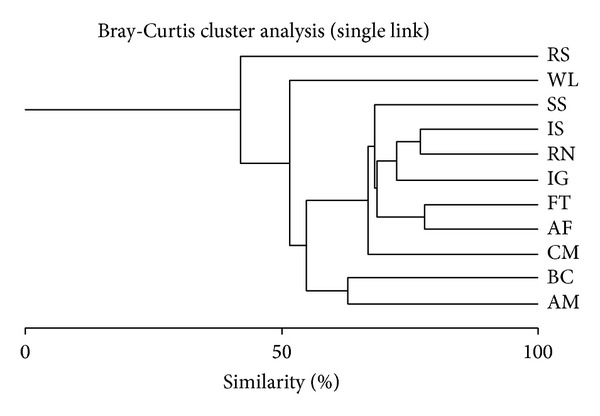
Species composition-based microlandscape clusters (AM: aerodrome; AG: agrofields; BC; business centers; CM; cemeteries; FT: forests; IS: informal settlements; IG: institutional grounds; RN: residential neighborhoods; RS: riversides; SS: streamside; WL: wasteland).

Of the 94 bird species recorded in Musanze city, 45 species were shared by both open fields and built-up landscapes. In terms of species composition found in each microlandscape type, the riverside landscape was the most different from the remaining microlandscapes. Residential neighborhoods and informal settlements were most similar. The differences between the landscape types in terms of species composition, illustrated by the increasing distance from the root to the point where a given branch stems, were as follows: riverside < aerodrome < streamside < wasteland < agrofields < cemeteries < forest < institutional and business center < informal settlements and residences (Figure 11).

### 3.5. Bird Diversity in Musanze City and Nearby Protected Areas

**Figure 12:**
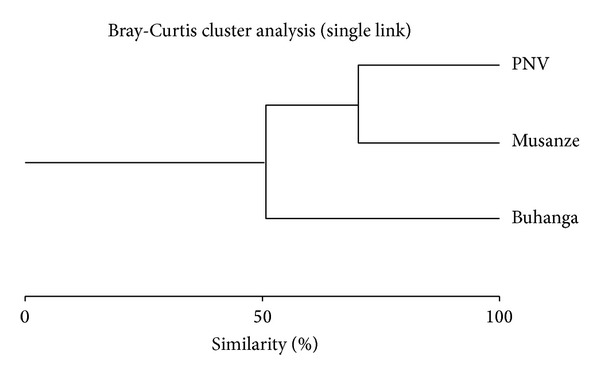
Musanze city, PNV, and Buhanga eco-park bird species composition.

In addition to the internal comparison, the level of similarity between Musanze and its nearby protected areas (PNV and Buhanga eco-park) revealed important similarities between protected and urban habitats in terms of bird diversity (Figure 12). The total number of bird species in Musanze city was 94, 105 in PNV, and 81 in the Buhanga eco-park. Of the 94 bird species recorded in Musanze, 32 were also present in Buhanga eco-park: 51 in the PNV and 21 were found in both the Buhanga eco-park and in PNV. Moreover, we found only one endemic bird species, the Ruwenzori Double-collared Sunbird (*Cinnyris stuhlmanni*), in Musanze, unlike the three endemic bird species present in Buhanga eco-park and the fifteen in the PNV. This Albertine Rift endemic species is also present in the PNV; however, it is absent from the Buhanga eco-park.

In the comparison of the bird species found in the three sites, Musanze city is unique in the number of migratory bird species observed. Specifically, seven migratory species were recorded in Musanze city, while only two are known to exist in the PNV and none have been recorded in the Buhanga eco-park (Table 4). Musanze's microlandscapes that were visited by migratory birds include the riversides of Mukungwa River, informal settlements, forests, business centers, and residential neighborhoods.

The majority of species in Musanze city were native, such that exotic bird species accounted for only 4.3% of the total number of bird species observed (*Ficedula albicollis, Ploceus cucullatus, Serinus canicollis, *and* Serinus citrinelloides*) (Table 5). Up to 6.4% of the species observed had no breeding opportunities within the study area (*Actitis hypoleucos, Aquila pomarina, Merops apiaster, Muscicapa striata, Phylloscopus trochilus, *and* Sylvia borin*).

## 4. Discussion

Our study of bird diversity in Musanze city has revealed that current wildlife conservation efforts do not coincide with areas of the highest bird abundance and species diversity, which exist in urban areas. Bird species richness in the urban areas of Musanze is greater than that of Buhanga eco-park and similar to that of the PNV, two surrounding natural habitats that are currently receiving the majority of conservation efforts. Furthermore, species of high ecological importance and therefore high conservation concern, such as endemic and migratory species, were recorded across the city landscapes. This study therefore indicates the importance of protecting not only the natural habitats of native wildlife but also urban areas where birds in particular are more commonly existing.

### 4.1. Species Richness

The unique, endemic species found in Musanze city, the Ruwenzori Double-collared Sunbird, has previously been observed in the VPN. This species is known to live in the high altitude habitats of the Albertine Rift [24]. It is therefore not surprising that it was found in urban landscapes of Musanze city, which is located at a high altitude of 1850 m [26]. Stevenson and Fanshawe [9] suggest that the sunbird's most preferred habitats are flowering bushes of forest edges, and it may be unusual that the endemic species was observed foraging on* Markhamia lutea *trees within open fields of Musanze city in this study. Our study suggests that the species distribution of the Ruwenzori Double-collared Sunbird is not restricted to its natural habitat in the Albertine Rift, as it can also exist in urban landscapes. This species may therefore be able to adapt to human-dominated landscapes and it is likely that it may forage on vegetation such as urban garden trees. Our study suggests that the sunbird is capable of adapting its feeding preferences to the available plants in city landscapes, potentially as a response to loss of its natural habitat caused by park reductions [27].

The seven migrant bird species found in the study site were regular September-April visitors from Palearctic regions. Two of them, the Red-chested Cuckoo (*Cuculus solitarius*) and the European Bee-eater (*Merops apiaster*), had previously been sighted in the gallery forests of the Rwandan's Eastern Province [28] and in the PNV [24]. The Common Sandpiper, Spotted Flycatcher, and Willow Warbler have also been reported to visit Rwanda (http://www.rwandabirding.org, 02.09.2012) and Musanze city but have never been sighted in the Lava Plain or in the Volcano Range of Rwanda. The remaining two migrants, the Garden Warbler (*Sylvia borin*) and the Lesser Spotted Eagle (*Aquila pomarina*), have been reported in Rwanda for the first time as a result of this study. Importantly, the seven migrant species have never been reported to occur in urban environments of Rwanda and only two had been reported to visit its highlands [24]. However, the African Pitta (*Pitta angolensis*), which is an intra-African breeding migrant bird sighted in the Buhanga eco-park in 2006 and 2008 [29], was not recorded in this study. The findings of our study encourage the development of bird watching activities in Musanze city, which may be of particular interest to international tourists due to the high number of urban-living, and therefore easily observed, unique bird species.

#### 4.1.1. Number of Species by Macrolandscape Types

The hypothesis that bird species inhabit open fields more than built-up areas was not supported in this study. This may be due to the fact that some built-up areas, including institutional grounds, residential neighborhoods, and informal settlements, are specifically designed with plant communities to attract birds [30]. These communities often include a number of trees with a substantial canopy, as well as ornamental plants, such as vegetation that exists as bushes. Alternatively, some microlandscapes within open fields have a low number of species such as the Musanze aerodrome, which is a grassland habitat and is regularly maintained to facilitate airplane landing. This habitat offers little feeding opportunities to wildlife and is an unsuitable habitat for most bird species as it may, for instance, expose small-sized birds to predators [31].

#### 4.1.2. Number of Species by Microlandscape Types

In support of Koellner et al. [32], different landscapes in Musanze were correlated with differences in bird species richness. The institutional grounds had a relatively high species richness, perhaps due to a high variety of plant assemblages. Similarly, the high number of bird species found in residential neighborhoods and informal settlements can be explained by the diversity of plant resources in these areas. These plant resources include nectar-producing flowers, such as banana plants that attract sunbirds, fruit-bearing trees, and shrubs, such as guava plants, that frugivorous species can feed on. In addition, the high concentrations of domestic wastes (such as residues, food disposable, and waste-water) in the homestead environment offer a unique opportunity to predators, such as insectivorous birds that feed on small mammals (e.g., mice, frogs) and insects (e.g., flies, mosquitoes), and are common to this type of microhabitats. Domestic waste may also attract scavenger species. Finally, informal settlements that are surrounded by a mosaic of vegetation types offer many opportunities for bird foraging and nesting [30]. This diversity in urban landscape vegetation structure and type may therefore reflect the vast diversity of bird species in urban areas, which has not previously been investigated in Rwanda.

### 4.2. Species Relative Abundance

#### 4.2.1. Number of Individuals by Macrolandscape Types

The nonsignificant effect of macrolandscapes on the total number of birds in an area may be linked to the fact that landscape type had no significant influence on bird species richness. More importantly, due to the historical nonformal nature of urban design in Musanze both macrolandscapes appeared to have similar types and levels of bird-exploitable resources, including appropriate plant coverage and sites for birds to nest [33].

#### 4.2.2. Number of Individuals by Microlandscape Types

Cemeteries, riverside landscapes, and wastelands did not differ in the total number of birds in comparison to residential neighborhoods (Table 3). This may be attributed to the fact that these four landscapes appear to be intrinsically heterogeneous, suggesting that there may be possible confounding factors that have overshadowed the expected relationship between microlandscapes and relative bird abundance. For instance, wastelands attract many scavengers and raptors, cemeteries and agrofields provide appropriate plant assemblages for seed eaters, and riversides offer easy water access and shelter for aquatic bird species [4]. Therefore, the abundance of birds across the different microlandscapes of Musanze is relatively uniform due to the fact that each landscape offers a range of suitable habitats for a range of bird species.

#### 4.2.3. Most Represented Species in Musanze City

The Pied Crow (*Corvus albus*) was the most common species in Musanze and seemed to be the most adapted to human-dominated landscapes (Figure 7). For instance, unlike many other bird species in the area the Pied Crow was observed close to many human facilities such food markets, abattoirs, wastelands, domestic yards, and croplands. In addition, the Pied Crow was found in high abundance where its food sources, including small reptiles, small mammals, grain, peanuts, carrion, scraps of human food, fruits, insects, and other small invertebrates, were most available [9]. To a lesser extent, the urban habitat appeared to favor the Grey-headed Sparrow (*Passer griseus*). The occurrence of the Grey-headed Sparrow was associated with open woodlands and human settlements, where this species can easily access required food and habitat resources. The third most represented species was the Streaky Seedeater (*Serinus striolatus*), which is a common bird of highlands and is generally found above 1300 m in gardens and cultivated areas, woodland edges, heath, and scrub vegetation areas. The Streaky Seedeater is therefore adept at living in human altered landscapes, which is not unlike the Black Kite (*Milvus migrans*), which was also common to densely human-populated areas. These human-used landscapes are also a popular habitat of the Black Kite's prey, which includes small birds, bats, and rodents. In particular, our study revealed the common occurrence of the Black Kite circling near the abattoir and scavenging in the city's wastelands.

### 4.3. Feeding Guild Categories

#### 4.3.1. Feeding Guild Categories by Macrolandscape Types

Seed-eating birds were the best represented guild in Musanze (Figure 8). This may be partially due to the fact that data collection was completed during a maize-harvesting period. During this period, urban fields have an exceptionally large abundance of seeds that can be exploited by birds and may have therefore selectively attracted seed-eating species [34]. It was also observed that seed-eating species fed from a range of fruits. According to Holland and DeAngelis [35], this habit allows seed-eating species to contribute to plant reproduction through pollination. Insectivorous species were also prominent in Musanze's urban landscapes. Similarly to the findings of Austin and Smith [5], insectivores were most abundant in insect-rich landscapes including streamside areas, forests, and informal settlements.

#### 4.3.2. Feeding Guild Categories by Microlandscapes

**Figure 13:**
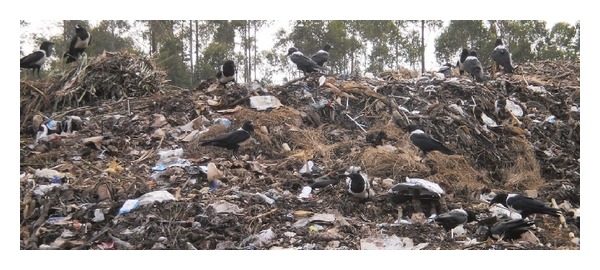
Abundance of Pied Crows in a landfill within Musanze city (Photo Author, 2012).

Although seedeaters, especially the Grey-headed Sparrow (*Passer griseus*), dominated almost all microlandscapes, it is important to consider that in wastelands scavengers were the most abundant (Figure 13). This finding is likely due to the fact that different microlandscapes are more appropriate for some species more than others, especially those with different feeding habits.

Our study confirms that bird species found in Musanze city, like urban-living bird species in other cities, contribute to biomass recycling [36]. Interestingly, although there were only a few species of scavenging birds in Musanze, their abundance was high enough to compensate for their low species richness in order to make a notable biomass recycling impact to city dwellers. This was particularly true for the Pied Crow (*Corvus albus*), a species whose abundance amounted to more than seven times the average number of individuals per species, which was almost 10% of the total number of birds observed.

### 4.4. Species Diversity

#### 4.4.1. Alpha Diversity

The bird diversity found in built-up areas was comparable to the bird diversity in urban open fields in Musanze city (Figure 9(a)). This finding provides support for the increasingly recognized idea that bird diversity in urban areas can be as high as that in natural environments [37, 38]. Previous research suggests that this may be due to a relatively high number of nonnative species present in cities as well as the heterogeneous habitat that urban landscapes provide [39], which was not supported in this study, perhaps because of close proximity of the study area to a network of natural environments (PNV, Gishwati forest, and Buhanga eco-park; Rugezi, Mukungwa, and Bihinga wetlands; and Karago, Bulera, and Ruhondo lakes). Moreover, Luck [40] suggested that there is a positive correlation between human population density and bird species richness. This relationship was demonstrated in our study, as species such as the Pied Crow were found to be most abundant throughout urban landscapes and therefore well adapted to an environment where high human densities exist. Interestingly, even the Albertine Rift endemic species, the Ruwenzori Double-collared Sunbird, was sighted foraging in human populated areas on garden plants.

Overall, residential neighborhoods, institutional grounds, and informal settlements can be considered as the major bird hotspots in Musanze city (Figure 9(b)). These three landscapes share a great floristic diversity, providing many important stimuli for bird life (such as fruits, seeds, nectar, domestic residues, insects, small mammals, reptiles, and amphibians) [30]. Finally, contrary to rural areas and natural forests that undergo seasonal shortages of resources [3], cities do not experience strong seasonal variation due to a continuous supply of recourses from remote areas.

#### 4.4.2. Species Similarity

The macrolandscapes all appeared to have similar bird species composition, which may be due to landscape heterogeneity [39]. This heterogeneity is likely a result of the lack of historical professional landscape planning and design, leaving a mosaic landscape structure in both built-up and open fields instead of a uniform landscape specialization. Our results suggest that residential areas and informal settlements were the most similar in terms of species composition. These landscapes ultimately function to host family life. The only difference between residential areas and informal settlements resides in their socioeconomic status, which is reflected in the landscape structure. However, both landscapes are more similar to each other than to other landscapes. Furthermore, the species composition of cemeteries was similar to both forests and some of the built-up microlandscapes, which may be attributed to the fact that these areas are all surrounded by trees. Additionally, cemeteries are characterized by low human disturbance, which may compare to that of forests and some urban landscapes. These landscapes all harbor a wide variety of flowering plants, which in the case of cemeteries are mainly planted by bereaved ones. Moreover, the vegetation of graveyards includes a range of regeneration stages resulting in a wide range of habitats, from the vegetation-free areas of newly dug graves to the reconstructed thickets around old graves [41]. Finally, riverside landscapes emerged as the most specialized landscape type as expected, with many distinctive species observed in this landscape only. Almost all the species found in riverside areas were specialists of wetlands and were not found elsewhere. As a result, it was the most dissimilar to all other microlandscapes.

The high degree of similarity in terms of bird diversity between Musanze city and the protected natural habitats nearby provide further support that wildlife does not exclusively inhabit protected areas [33, 37, 42]. Although the two natural habitats are at eight km from Musanze city, more than half of the species found in the city were also found in VPN. The lower degree of similarity between Musanze and Buhanga eco-park may be linked to the relative geographic location of the two sites, as Musanze city is almost half way between the two protected areas. This finding may also be attributed to the fact that the diversity of microhabitats within Buhanga eco-park is lower than that of Musanze and VPN [13], which may be due to the relatively small size of Buhanga eco-park. Interestingly, the species found in more than one landscape type were also recorded both inside and beyond the city limits. Conversely,* Ficedula albicollis* was recorded in only one landscape type (cemeteries) and was absent in both PNV and Buhanga eco-park.

## 5. Conclusions

This study contributes to the knowledge of bird diversity in urban landscapes and provides the most recent status of bird diversity in Musanze city. Our findings confirm that bird diversity in urban areas, such as a densely populated city, can be as great as that in surrounding natural forests. Specifically, the total number of bird species in Musanze city was very similar to that of the nearest protected forests, Volcanoes National Park, and Buhanga eco-park. Endemic and migratory birds were also found in Musanze city. One Albertine Rift endemic bird species, the Ruwenzori Double-collared Sunbird, was observed in Musanze during this study, which has not been previously recorded. Similarly, three migratory birds were found in Musanze region for the first time: the Common Sandpiper, the Spotted Flycatcher, and the Willow Warbler. In addition, two bird species were observed that have not been previously reported in Rwanda: the Garden Warbler (*Sylvia borin*) and the Lesser Spotted Eagle (*Aquila pomarina*). The effect of landscape types on bird species richness and relative abundance was also illustrated. Residential neighborhoods, institutional grounds, and informal settlements had the highest species diversity indices and rank/abundance relative to the rest of the microlandscapes in this study. Riverside landscapes emerged with the most specialized bird fauna, including species that are restricted to wetland environments and water bodies. This study confirmed that scavengers in Musanze city contribute more to biomass recycling than any other bird feeding groups. Our study provides a current and useful reference for urban decision makers and confirms the existence of a great diversity of wildlife within cities. We suggest that the development and implementation of city land-use plans should consider this biodiversity, especially when villages and cities are in close proximity of protected areas or natural reserves. Finally, it may be important that botanical gardens and public parks are included in the master plan of the city, as our study indicates that their design requires an understanding of biodiversity, in particular of avian populations.

## Figures and Tables

**Table 1:** Distribution of plots across microlandscape types and administrative sectors.

Landscape types	Category	Sectors	Number of plots
Aerodrome	Open field	Muhoza	2
Agrofields	Open field	Muhoza, Cyuve	5
Business centers	Built-up	Musanze, Muhoza, Cyuve	5
Cemeteries	Open field	Muhoza	2
Forests	Open field	Musanze, Muhoza, Cyuve	5
Institutional grounds	Built-up	Musanze, Muhoza	5
Residential neighborhood	Built-up	Muhoza	5
Riversides	Open field	Muhoza	2
Informal settlements	Built-up	Kimonyi, Muhoza	5
Streamsides	Open field	Musanze	2
Wastelands	Open field	Muhoza, Cyuve	2

**Table 2:** LMM output showing parameter estimates of microlandscape types on the number of bird species.

Landscape types	Std. error	*t* value	*P* value
Intercept	0.74	15.338	<0.001
Aerodrome	1.39	−5.502	<0.001
Agrofield	1.05	−3.330	<0.01
Business centers	1.05	−6.374	<0.001
Cemeteries	1.39	−1.816	0.069
Forests	1.05	−3.473	<0.01
Institutional ground	1.05	−2.807	<0.01
Riversides	1.39	−0.827	0.408
Informal settlements	1.05	−3.330	<0.01
Streamside	1.39	−2.445	<0.05
Wastelands	1.39	−2.625	<0.01

**Table 3:** LMM output showing parameter estimates of microlandscape types on the number of bird individuals.

Landscape types	Std. error	*t* value	*P* value
Intercept	0.12	27.250	<0.001
Aerodrome	0.224	−4.970	<0.001
Agrofield	0.169	−2.330	<0.05
Business centers	0.169	−6.646	<0.001
Cemeteries	0.224	−1.919	0.055
Forests	0.169	−3.615	<0.001
Institutional ground	0.169	−2.229	<0.05
Riversides	0.224	−0.16	0.873
Informal settlements	0.169	−2.899	<0.01
Streamside	0.224	−2.029	<0.05
Wastelands	0.224	0.173	0.860

**Table 4:** Migratory birds in Musanze city, PNV, and Buhanga eco-park.

Common name	Scientific name	Musanze city	Protected areas	
			PNV	Buhanga eco-park
Common Sandpiper	*Actitis hypoleucos *	*✓*		
European Bee-eater	*Merops apiaster *	*✓*	*✓*	
Garden Warbler	*Sylvia borin *	*✓*		
Lesser-spotted Eagle	*Aquila pomarina *	*✓*		
Red-chested Cuckoo	*Cuculus solitarius *	*✓*	*✓*	
Spotted Flycatcher	*Muscicapa striata *	*✓*		
Willow Warbler	*Phylloscopus trochilus *	*✓*		

**Table 5:** Exotic birds in Musanze city, PNV, and Buhanga eco-park.

Common name	Scientific name	Musanze city	Protected areas	
			PNV	Buhanga eco-park
Collared Flycatcher	*Ficedula albicollis *	*✓*		
Black-headed Weaver	*Ploceus cucullatus *	*✓*	*✓*	*✓*
Yellow-crowned Canary	*Serinus canicollis *	*✓*	*✓*	*✓*
Abyssinian Citril	*Serinus citrinelloides *	*✓*	*✓*	*✓*
